# Winning Fights Induces Hyperaggression via the Action of the Biogenic Amine Octopamine in Crickets

**DOI:** 10.1371/journal.pone.0028891

**Published:** 2011-12-21

**Authors:** Jan Rillich, Paul Anthony Stevenson

**Affiliations:** 1 Institute for Neurobiologie, Freie University of Berlin, Berlin, Germany; 2 Institute for Biologie, University of Leipzig, Leipzig, Germany; University of Plymouth, United Kingdom

## Abstract

Winning an agonistic interaction against a conspecific is known to heighten aggressiveness, but the underlying events and mechanism are poorly understood. We quantified the effect of experiencing successive wins on aggression in adult male crickets (Gryllus bimaculatus) by staging knockout tournaments and investigated its dependence on biogenic amines by treatment with amine receptor antagonists. For an inter-fight interval of 5 min, fights between winners escalated to higher levels of aggression and lasted significantly longer than the preceding round. This winner effect is transient, and no longer evident for an inter-fight interval of 20 min, indicating that it does not result from selecting individuals that were hyper-aggressive from the outset. A winner effect was also evident in crickets that experienced wins without physical exertion, or that engaged in fights that were interrupted before a win was experienced. Finally, the winner effect was abolished by prior treatment with epinastine, a highly selective octopamine receptor blocker, but not by propranolol, a ß-adrenergic receptor antagonist, nor by yohimbine, an insect tyramine receptor blocker nor by fluphenazine an insect dopamine-receptor blocker. Taken together our study in the cricket indicates that the physical exertion of fighting, together with some rewarding aspect of the actual winning experience, leads to a transient increase in aggressive motivation via activation of the octopaminergic system, the invertebrate equivalent to the adrenergic system of vertebrates.

## Introduction

Throughout the Animal Kingdom conflict between individuals of the same species radically changes the contestants' future behaviour such that losers become less and winners more aggressive and more likely to win subsequent encounters, even against novel opponents (for review see [1]). A wide variety of hypotheses offer explanations for the occurrence of these winner and loser effects [1]–[6], but comparatively little is known of their proximate causes.

With respect to the winner effect, the most recent experimental data implicate androgens as physiological mediators in both fish [7] and mice [8]. This is in line with the challenge hypothesis, according to which social challenges raise testosterone levels to facilitate competitive behaviour, including aggression (for review see [5], [6]). It has, however, been argued, that winner and loser effects are probably “not merely by-products of hormonal mechanisms that regulate agonistic behaviour, because similar winner/loser effects exist in vertebrates and invertebrates which have significantly different physiological mechanisms to regulate agonistic behaviour” [3]. In insects, associations between levels of juvenile hormone, social interactions and aggressive behaviour have been put forward as support for the challenge hypothesis in invertebrates [9]–[11]. However, neither ablation of the corpora allata [12], which secrete juvenile hormone, nor juvenile hormone supplementation have been found to actually influence aggression [13], so that the challenge hypothesis, as applied to testosterone and vertebrates, does not yet have an analogous model for juvenile hormone and insects [13].

Our studies of aggression in crickets have shown that experiences as diverse as flying, and residency lead to enhanced expression of aggression, and nullify the loser effect via a mechanism involving release of the biogenic amine octopamine [14]–[16]. The endogenous monoamine octopamine is an analogue of norepinephrine (noradrenaline], which acts as a neurotransmitter, neuromodulator and neurohormone in insects and other protostomes [17], and has equivalent functions to those of the adrenergic system in vertebrates [18]–[20]. In this paper we investigate the influence of selected amine-receptor antagonists on the winner effect in male Mediterranean field crickets (Gryllus bimaculatus). Previous studies on crickets [21]–[23], as well as other insects and arthropods [24]–[26], have shown that an experience of dominance increases the probability of exhibiting aggressiveness or winning in subsequent encounters against a loser or non-experienced opponent. As fights are usually decided by the action of subordinates [27], we chose to quantify the winner effect from the performances of winners matched against each other in knockout tournaments. Our data illustrate that the experience of winning transiently enhances aggressiveness in crickets via a mechanism dependent on the action of the octopaminergic neuromodulator/hormonal system.

## Results

### General observations

As claimed elsewhere [21], crickets that had won a previous encounter were more likely to win against a fight inexperienced conspecific (73% of 49 encounters, Chi^2^-test p = 0.019). Moreover, these winners usually initiated the contest, for example by being the first to spread their mandibles (76%, Chi^2^-test p = 0.007). In the following we further quantified the winner effect in crickets by evaluating aggressive escalation and fight duration in knockout tournaments.

### Knockout tournaments - the winner effect

**Figure 1:**
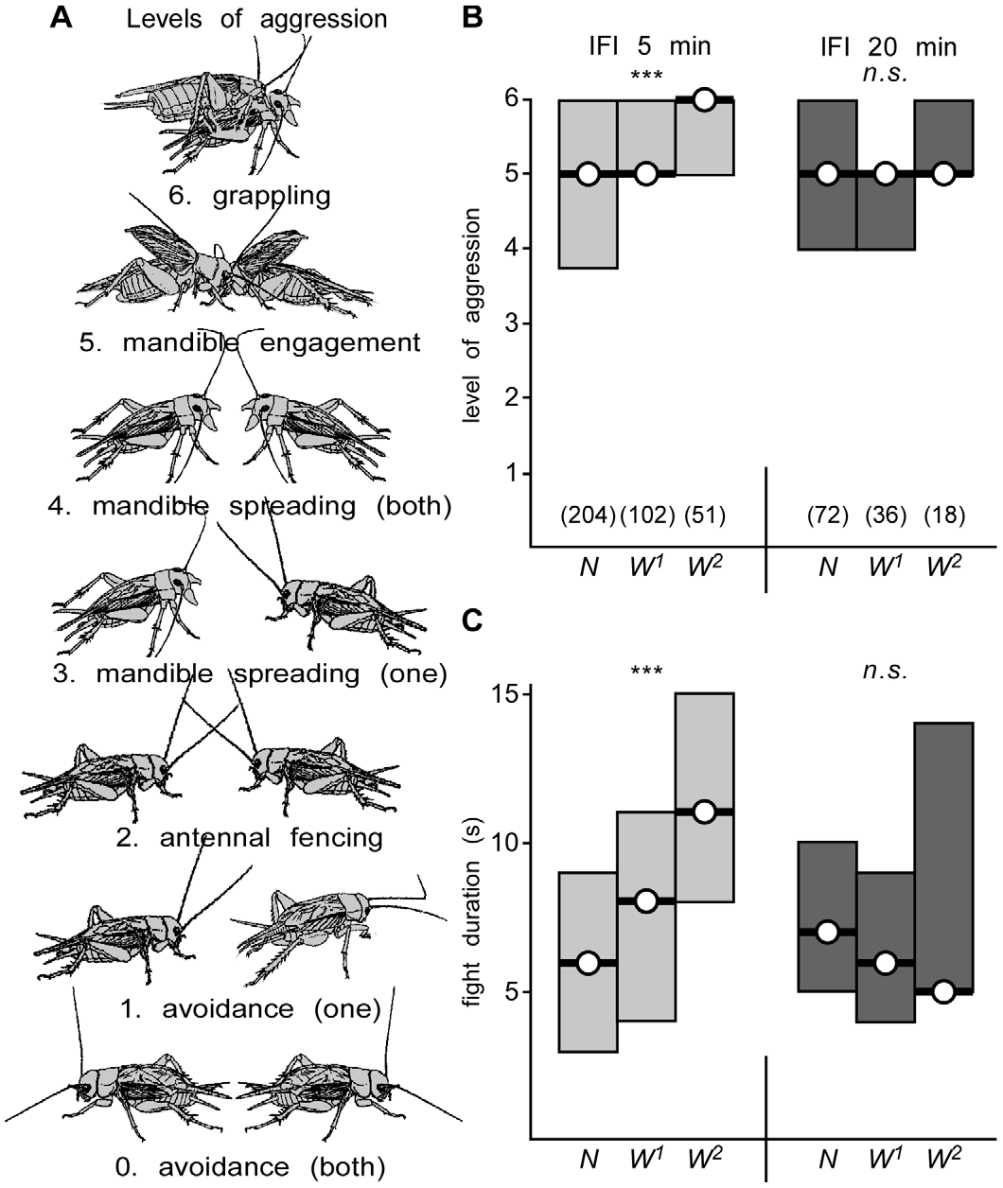
Stereotyped fighting and the winner effect in crickets. A Pictograms illustrating the escalating levels of aggression that characterize cricket fights (adopted from [15]): Level 0 mutual avoidance: non-aggressive interaction. Level 1 pre-established dominance: one cricket attacks, the other retreats. This level is in accordance with the avoiding behaviour of losers. Level 2 antennal fencing: the two crickets fence with their antennae. Level 3 mandible spreading (unilateral): one cricket displays broadly spread mandibles. Level 4 mandible spreading (bilateral): both crickets display their spread mandibles. Level 5 mandible engagement: the mandibles of both contestants interlock. Level 6 grappling: all-out fighting involving repetitive biting, mandible pushing, and opponent flipping. B, C Bar graphs giving the level and respectively duration of aggression for encounters between pairs of male, weight matched, fight-inexperienced crickets (naive, N), winners of one previous encounter (W^1^), winners of two previous encounters (W^2^) for inter-fight intervals (IFI) of 5 min (left side, grey bars) and 20 min (right side, dark bars). Numbers in parenthesis in (B) give the number of contests for each round, circles with bar the median, boxes the interquartile range and asterisks significant differences between all rounds in the tournament (Kruskal-Wallis one way variance test, *** p<0.001, n.s. not significant).

Inter-fight interval 5 min. Fights between fight inexperienced (naive, N) weight-matched adult male crickets correspond in all major respects to previous descriptions [15], [16]. Their encounters usually involved physical contact (median level of aggression 5, I.Q.R. 3.75–6, n = 204, Fig. 1) and lasted some 6 s (median, I.Q.R. 3–9). To evaluate the effect of winning, we matched the winners of initial fights against each other in a second round (W^1^), and subsequently the new winners against each other in a 3rd round (W^2^). In a tournament with the inter-fight interval (IFI) set to 5 min the fights escalated to significantly higher levels, and lasted longer with each round (Kruskal Wallis test: p-level<0.001, p-duration<0.001; W^1^: median level 5, I.Q.R. 5–6, median duration 8 s, I.Q.R. 4–11, n = 102; W^2^: median level 6, I.Q.R. 5–6, median duration 11 s, I.Q.R. 8–15, n = 51). At the 3rd round for example nearly all fights escalated to the highest level (6, grappling) and lasted twice as long as fights at the initial encounter (U test: p-level<0.001, p-duration<0.001).

Inter-fight interval 20 min. The above described winner effect appeared to be transient. When the IFI was extended to 20 min, successive winning experiences had no significant influence on aggressiveness exhibited in the tournaments (Kruskal Wallis test: p-level = 0.44, p-duration = 0.77; N: median level 5, I.Q.R. 4–6, median duration 7 s, I.Q.R. 5–10, n = 72; W^1^: median level 5, I.Q.R. 4–5, median duration 6 s, I.Q.R. 4–9 m; n = 36; W^2^: median level 5, I.Q.R. 5–6, median duration 5 s. I.Q.R. 5–14, n = 18, Fig. 1). For example, fights of the third round (W^2^) were not significantly different to those between naïve contestants (U test: p-level = 0.304, p-duration = 0.75) but significantly less aggressive (U test: p-level = 0.003) and shorter (U test: p-duration = 0.04) than third round fights between winners in the tournament with an IFI of 5 min.

### Effect of aminergic blockers

**Figure 2:**
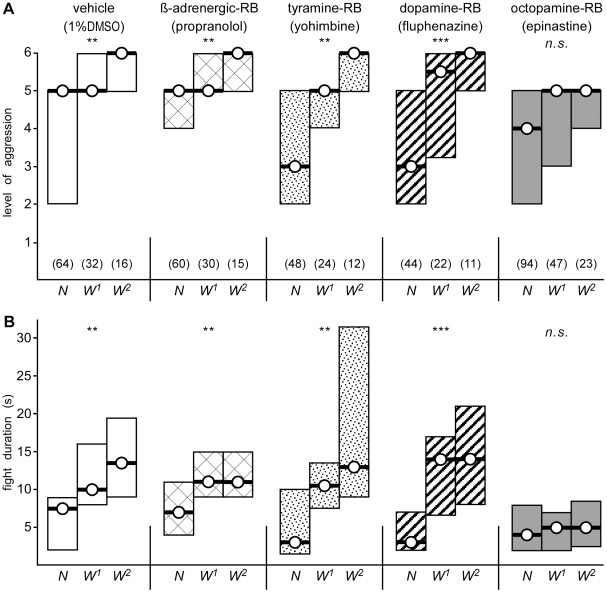
Effect of amine-receptor antagonists on the winner effect. A, B Bar graphs giving the level, and respectively duration of aggression for encounters between pairs of male, weight matched, fight-inexperienced crickets (naive, N), winners of one previous encounter (W^1^), winners of two previous encounters (W^2^) for inter-fight intervals (IFI) of 5 min following treatment with (from left to right): vehicle (white bars), propranolol (cross-hatched bars bar), yohimbine (stippled bars), fluphenazine (bold-hatched bars) or epinastine (dark grey bars). Numbers in parenthesis in (A) give the number of contests for each round, circles with bar the median, boxes the interquartile range and asterisks significant differences between all rounds for each tournament (Kruskal-Wallis one way variance test, ** p<0.01, *** p<0.001, n.s. not significant).

To test whether the winner effect depends on the action of biogenic amines, we staged tournaments with an IFI of 5 min as described above, but in which the animals were pre-treated 1–2 hours prior to fighting with a spectrum of neurochemicals known to block selected amine-receptors in insects. Our findings are summarized in Fig. 2 and Table 1. Fights between fight inexperienced (N) crickets injected with vehicle (20 µl, 2% aqueous dimethyl sulphoxide, DMSO) as a control, did not differ to those of untreated crickets with respect to the level of aggression and duration of the fight (U tests: p-level>0.05, p-duration>0.05 for all groups, Fig. 2). Moreover, with each successive round in the tournament the fights escalated to significantly higher levels, and lasted longer (Kruskal Wallis test: p-level = 0.013, p-duration = 0.0036; N: median level 5, I.Q.R. 2–5, median duration 7.5 s, I.Q.R. 2–9, n = 64; W^1^: median level 5, I.Q.R. 5–6, median duration 10 s, I.Q.R. 8–16, n = 32; W^2^: median level 6, I.Q.R. 5–6, median duration 13.5 s, I.Q.R. 9–19.5, n = 16). Similarly, with the exception of epinastine none of the amine receptor blockers appear to influence aggressiveness as measured here. For example, interactions between fight-inexperienced crickets treated with propranolol (a vertebrate β-adrenoceptor blocker with low affinity for insect aminergic receptors), yohimbine (a potent insect tyramine receptor blocker) or fluphenazine (an insect dopamine-receptor blocker) were not significantly different to those of vehicle-treated controls (U tests: p-levels and p-durations >0.05 for all groups). A winner effect was also clearly evident for each of these groups in that the level of aggression and fight duration increased significantly with each tournament round (Kruskal Wallis tests: propranolol p-level = 0.0093, p-duration = 0.0056; yohimbine p-level = 0.0078, p-duration = 0.0076; fluphenazine p-level<0.001, p-duration<0.001; see Table 1). Supporting this, the level of aggression and fight duration of 3rd round fights (W^2^) of crickets fights treated with either a β-adrenoceptor-, tyramine- or dopamine-receptor blocker were not significantly different to vehicle treated controls (U tests: p-levels and p-durations >0.05 for all groups).

**Table 1:** Numerical summary of the aggressiveness exhibited by crickets treated with amine-receptor blockers in knockout tournaments.

	N vs. N		W1 vs. W1		W2 vs. W2		p - values	
Test group	level	duration, s	level	duration, s	level	duration, s	level	duration
vehicle	5, 2–5	7.5, 2–9	5, 5–6	10, 8–16	6, 5–6	13.5, 9–19.5	0.013	0.0036
ß-blocker	5, 4–5	7, 4–11	5, 5–6	11, 9–15	6, 5–6	11, 9–15	0.0093	0.0056
tyramine-RB	3, 2–5	3, 1.5–9	5, 4–5	10.5, 7.5–13.5	6, 5–6	13, 9–31.5	0.0078	0.0076
dopamine-RB	3, 2–5	3, 2–7	5.5, 3.25–6	14, 6.5–17	6, 5–6	14, 8–21	<0.001	<0.001
octopamine-RB	4, 2–5	4, 2–8	5, 3–5	5, 2–7	5, 4–5	5, 2.5–8.5	0.25	0.80

Test group: vehicle (DMSO in ringer), ß-blocker (propranolol), tyramine-, dopamine-, octopamine-RB (the receptor blockers yohimbine, fluphenazine and epinastine respectively). N vs. N: first round, naïve, fight inexperienced crickets; W1 vs. W1: second round, winners of first round; W2 vs. W2: third round, winners of second round. The median and interquartile range (IQR) is given for the level of aggression and total fight duration (inter-fight interval 5 min in each case) as well as p values for the tournaments from Kruskal Wallis analyses.

Contrasting the above, the winner effect was abolished in the tournament for crickets treated with the selective octopamine receptor blocker epinastine (Kruskal Wallis tests: p-level = 0.25, p-duration = 0.80, both statistically non-significant). Furthermore, even though fights between fight-inexperienced crickets (N) did not differ for the vehicle and octopamine-blocker tournaments (U tests: p-level = 0.20, p-duration = 0.08) the 3rd round fights (W^2^) for the octopamine-blocker group did not escalate as high and were significantly shorter than those of the vehicle treated controls (W^2^ epinastine: median level 5, I.Q.R. 4–5, median duration 5 s, I.Q.R. 2.5–8.5, n = 23, U tests: p-level<0.001, p-duration = 0.04).

### The winning experience

Two series of experiments were performed to determine whether the winner effect is primarily due to experiencing the retreat of adversaries, without actually fighting (winning without fighting), or to the physical experience of fighting, without experiencing an actual win (fighting without winning).

#### Winning without fighting

**Figure 3:**
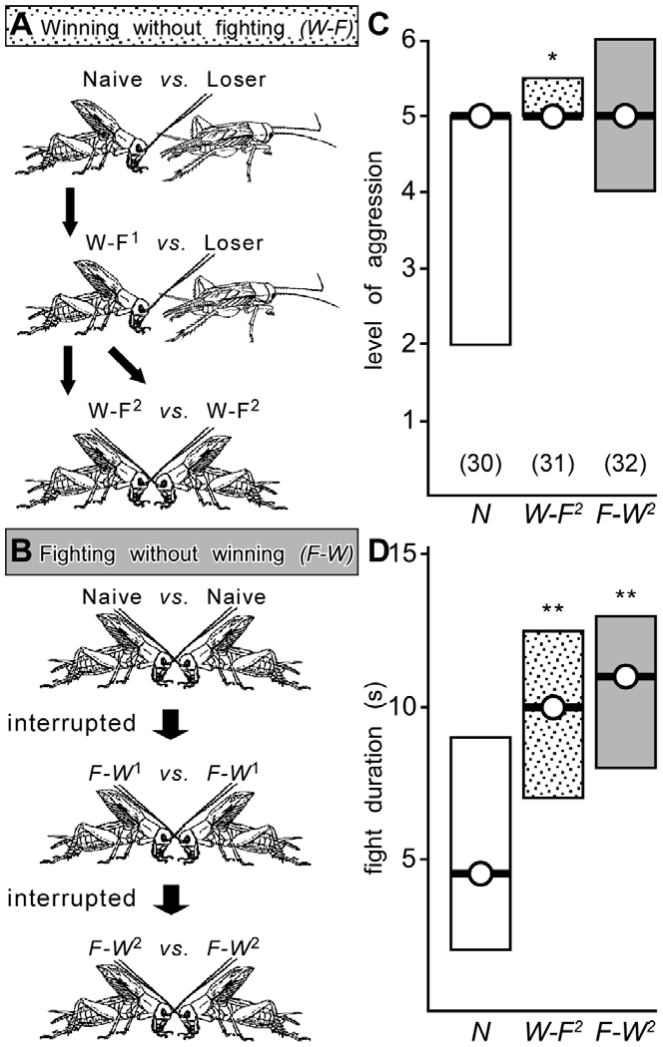
Influence of different winning experiences on subsequent aggression. A, B Pictograms illustrating the paradigms “winning without fighting” and respectively “fighting without winning”. C, D Bar graphs giving the level and respectively duration of aggression for encounters between pairs of male, weight matched crickets with different winning experiences, from left to right: control - no previous fighting experience (naive, N, white bars), “winning without fighting”, i.e. crickets that were tested following 2 successive encounters against non-aggressive losers (W-F^2^, stippled bar), “fighting without winning”, i.e. crickets that were tested following 2 successive fights that were interrupted before a win occurred (F-W^2^, grey bar). The inter-fight interval (IFI) was 5 min in all cases. Numbers in parenthesis in (C) give the number of contests for each round, circles with bar the median, boxes the interquartile range and asterisks significant differences between all rounds in the tournament (U test, * p<0.05, ** p<0.01).

In this experiment, initially fight inexperienced crickets were first matched twice consecutively (interval 5 min) with a cricket that had previously lost a fight and retreated without any aggressive interaction (pictogram Fig. 3A). The crickets that experienced retreating adversaries exhibited all behaviours typical for winners, including body jerks (100%, n = 62 individuals) and rival song production (42%; not significantly different to normal winners: body jerks 100%, rival song 53%, n = 30; Chi^2^-test: p>0.05). This experience of winning without fighting was alone sufficient to enhance aggression expressed in a subsequent test. Thus, fight-inexperienced winners paired against one other (W-F^2^) escalated to higher levels of aggression (median 5, I.Q.R. 5–5.5, n = 31, Fig. 3C) and fought longer (median duration 10 s, I.Q.R. 7–12.5, Fig. 3D) than inexperienced, naive crickets (median level 5, I.Q.R. 2–5, median duration 4.5 s, I.Q.R. 2–9, n = 30, U tests: p-level = 0.047, p-duration = 0.0041).

#### Fighting without winning

In this experiment, fight inexperienced crickets were matched against each other on two consecutive rounds, whereby the fights were manually interrupted after interacting for 4 s, before a winner was established (IFI, again 5 min, pictogram Fig. 3B). In these contests, the frequency of body jerk (31%, n = 64 individuals) and rival song (17%) were both significantly lower (Chi^2^-test: body jerks p>0.001, rival song p>0.001). Nonetheless, experiencing 2 consecutive, interrupted fights without winning was still sufficient to enhance aggression in a subsequent test. These win-inexperienced crickets fought each other (F-W^2^) significantly longer compared to naive crickets (median duration 11 s, I.Q.R. 8–13, n = 32, U test: p-duration = 0.0065, Fig. 3D). However, the level of aggression (median 5, I.Q.R. 4–6, Fig. 3C) was not significantly higher than the control group, but also not significantly different to the winning without fighting group. The data thus show that both physical fighting and observing an opponent retreat can enhance aggressiveness, although there is some indication that the latter experience may be somewhat more effective.

## Discussion

In this paper we evaluated the winner effect [1], [3] by staging tournaments between weight-matched adult male crickets. We demonstrate that hyper-aggressiveness resulting from repeated victory in these insects is dependent on the biogenic amine octopamine, the invertebrate analogue of norepinephrine.

Winning increased the level of aggression to which the crickets escalate during fighting, the total fight duration and the chance of winning against fight-inexperienced rivals. While this effect was clearly evident for an inter-fight interval of 5 min, winning had no significant effect on fighting when the interval was extended to 20 min. Hence, it cannot be simply due to selecting and matching individuals that were excessively aggressive from the outset [28]. According to our data, the winner effect lasts somewhat shorter than previously estimated in crickets (1–6 h, [22]), jumping spiders (1 h, [29]), sun fish (50–60 min, [30]), and is considerably shorter than in rodents (2–6 days, [8]). The winner effect in crickets is also far shorter than the loser-effect (see also [29]), which depresses aggression for hours after defeat [31], indicating that the mechanisms underlying winner and loser effects differ, as in cichlid fish [7]. On the other hand, the similar time courses of the enhancing effect of winning (this paper) and residency on aggression [16] suggest a common underlying mechanism for these two phenomena.

Indeed, as earlier demonstrated for flight [15] and residency [16], the winner effect was prohibited by treatment with epinastine, a highly selective blocker of insect neuronal octopamine receptors (cf. [32]), while other amine-receptor antagonists were ineffective. The latter included the vertebrate beta-adrenergic receptor blocker, propranolol, which has a low affinity for octopamine receptors [33], yohimbine, which blocks receptors for octopamine's metabolic precursor tyramine [19] and fluphenazine, an effective, but sub-type unspecific antagonist for dopamine receptors in crickets [34]. To our knowledge this is the first clear demonstration that a monoamine is involved in the mechanism that underlies the transient enhancing effect of repeated victory on aggression. Considering our findings that the aggression-enhancing effects of flying [14], [15], residency [16] and winning (this paper) are all octopamine dependent, we hypothesize, that all experiences leading to enhanced aggressiveness are likely to be mediated via activation of the octopaminergic system in crickets.

A question that still needs to be addressed is what elements of the entire behavioural experience of winning an aggressive encounter produce the winner effect? While fighting in crickets results in an almost 5 fold increase in the octopamine content of the haemolymph, this occurs irrespective of whether the animals won or lost the encounter [35]. This has two implications. Firstly, that activation of the octopaminergic system may not protect a cricket from losing. Supporting this idea, the pesticide chlordimeform, which binds almost irreversibly to octopamine receptors (cf. [18], [36]), has a prolonged enhancing effect on aggressiveness, and restores aggressiveness in losers, but cannot prohibit a defeat from actually occurring [15]. The second implication is that the winner effect may be due to elevated octopamine levels resulting alone from the physical exertion of fighting, without losing, rather than winning per se. Indeed, by staging fights between individuals that had previously participated in physical agonistic interactions that were interrupted before a conclusion was reached, we established that fighting without actually experiencing a win enhanced aggression in subsequent encounters. This is in line with our earlier finding that physical activities such as flying enhances aggressiveness in crickets [31] and that this effect is mediated by the amine octopamine [14], [15]. We thus suggest that the physical act of fighting may also promote aggression due to concomitant activation of the octopaminergic system. This is feasible considering that various locomotor behaviours including flying and walking as well as the resultant stimulation of dedicated proprioceptors activates selected sets of octopaminergic neurones (“DUM-cells”, [37], [38]).

On the other hand, fights between individuals that experienced an opponent retreating prior to any physical contact, established that winning without physically fighting was an equal, if not even more, effective aggression enhancing experience. A similar phenomenon is known in ants, where the mere exposure to an opponent, without the encounter escalating to a fight, increases the probability that it will display aggression in later encounters [39]. Furthermore, in humans merely watching a previous victory elevates levels of testosterone [40], a hormone with demonstrated promoting effects on aggression in rodents [41]. In mammals and other vertebrates, there are indications that an aggressive encounter may be evaluated as a rewarding experience, due to increased activity in dopaminergic pathways and increased androgen receptor expression in brain regions that mediate motivation and reward [8], [42], [43]. Interestingly, numerous studies in insects and other arthropods have shown that the amine octopamine, rather dopamine, plays a dominant role in mediating reward responses [44]. For example, octopamine enhances olfactory reward conditioning and memory retrieval in both honey bees and crickets [45], [34]. In honey bees, activating even a single octopaminergic neurone can substitute for the sucrose reward in an associative learning paradigm [46]. This neurone is member of a well known group of octopaminergic neurones in insects (DUM/VUM cells, [47], [48]), that has its soma in the suboesophageal ganglion and bilateral ascending projections that ramify in all major brain neuropils of the brain, including the mushroom bodies, a higher centre involved in olfactory processing, learning and memory. Interestingly, cells of this type occur in the same region of the nervous system that houses a subset of octopaminergic neurones that are required for the expression of aggression in Drosophila [49]. We therefore propose that, in addition to the physical exertion of fighting, some aspect of the event of actually winning, such as observing the opponent retreat, also represents a positive, rewarding experience that triggers octopamine release in the brain and thereby enhances aggression exhibited in subsequent encounters. This idea is in line with our previous findings that the experience of occupying a shelter, which like winning without fighting is also an essentially non-physical activity, transiently enhances aggression in crickets by an octopamine-dependent mechanism [16].

To what extent is the mechanism underlying the winner effect in insects comparable to that in mammals? While winner effects in vertebrates are generally longer and regulated primarily by androgens [7], [8], aminergic mechanisms may also be involved. In rodents, noradrenergic activation seems to be necessary for the expression of mammalian aggression [50] and repeated fighting lowers levels of serotonin, which depresses aggression in both mammals [51] and insects [52], while activating dopamine associated systems [53], [54] that are involved in mediating reward and the responses to many social stimuli (reviews: [43], [44]). Whether the activation of the catecholaminergic system is alone sufficient to induce winner effects in vertebrates, as shown here for octopamine in a model invertebrate, remains to be established.

In conclusion, our studies in the cricket indicate that the physical exertion of fighting, together with some rewarding aspect of the winning experience, leads to a transient increase in aggressive motivation via activation of the octopaminergic system. In addition to increasing our understanding of how concepts such as reward and motivation may be encoded in the nervous system of a presumably non-conscious animal, insects may be viable models for investigating reward associated hyper aggression.

## Materials and Methods

### Ethics statement

All treatments of the experimental animals (crickets, Gryllus bimaculatus - Insecta) complied with the Principles of Laboratory Animal Care and the German Law on the Protection of Animals (Deutsches Tierschutzgesetz).

### Experimental animals

Mature adult male Mediterranean field crickets, Gryllus bimaculatus (de Geer) were taken from a breeding stock maintained under constant standard conditions at Leipzig University (22–24°C, relative humidity 40–60%, 12 h∶ 12 h light∶ dark regime daily feeding on bran and fresh vegetables) and kept isolated in individual glass jars for at least 24 h prior to all experiments, which were performed during daylight hours, avoiding times when aggression tends to be depressed (just after midday and on generally dreary days; see [55]). Altogether the behaviour of 958 crickets was evaluated.

### Knockout tournaments

To evaluate the effects of winning we staged knockout tournaments between pairs of weight-matched, adult male crickets. Crickets with no previous fighting experience were first matched against each other (naive versus naive: N). Each contestant was placed at one end of a Perspex glass arena (16×9 cm) and separated from each other by an opaque grey, plastic dividing door. After leaving the animals for 2 min to adapt to the new situation, the dividing door was removed manually, following which the animals invariably contacted each other and exhibited fighting behaviour (cf. [15]). The winners of this first round were then matched against each other (W^1^), and the resulting winners matched against each other in the final round (W^2^). The interval between consecutive fights was 5 min for all tournaments excepting one, in which the inter-fight interval (IFI) was 20 min (see results). At least 8 pairs of crickets were tested in any one tournament session, and data were accumulated from experimental sessions performed on different days. The numbers of cricket pairs for each tournament is given in the results.

### Dissecting the winning experience

#### Winning without fighting

To test whether the winner effect depends alone on experiencing the retreat of adversaries, initially fight inexperienced crickets were matched twice consecutively against a subordinate cricket that previously lost a fight and retreated immediately, without fighting, when confronted. Crickets that experienced 2 such wins without fighting were then matched against each other (W-F^2^) to evaluate their aggressiveness (pictogram Fig. 3A).

#### Fighting without winning

To test whether the winner effect depends alone on the experience of actually fighting, initially fight inexperienced crickets were matched twice consecutively against each other, but here the fights were interrupted before either won by manually separating the contestants after a 4 s period of physical interaction. Pairs of crickets that experienced 2 such fights without winning were then matched against each other (F-W^2^) without interruption in a final encounter to evaluate their aggressiveness (pictogram Fig. 3B).

### Pharmacological treatments

Crickets were fastened dorsal side upwards onto a corkboard using commercial grade modelling clay and a small hole was punctured medially into the dorsal surface of the head capsule using a fine tungsten steel insect pin. Drugs were injected through the punctured hole in the near vicinity of the brain using a micro-syringe (Hamilton, Bonaduz, Switzerland) mounted on a micromanipulator (Bachhofer, Reutlingen, Germany). Groups of test crickets were injected with 20 µl of 20 mM solutions of amine receptor antagonists in 2% aqueous dimethylsulphoxide (DMSO; cf. [15]): the ß-adrenoceptor blocker propranolol, the tyramine receptor blocker yohimbine, the D1/D2 dopamine receptor blocker fluphenazine, or the selective octopamine receptor blocker epinastine. Control animals received the vehicle DMSO only. Unless stated otherwise, all drugs were obtained from Sigma Aldrich (Deisenhofen, Germany). Aggressive behaviour was then analysed within 1–2 hours after treatment in tournament contests (as above) between animals that received the same treatment. In each experimental session we run at least 3 separate tournaments, with 2 test groups and one control group, comprising 12 pairs of crickets each. Data was accumulated from multiple experimental sessions. The numbers of cricket pairs for the test and control tournaments is given for each test group in the results.

### Data analysis

The intensity of aggressive interactions were scored on a scale of 0–6 (cf. [15] and Fig. 1A) denoting the level to which a fight escalates before the winner is established by the retreat of one contestant: Level 0: mutual avoidance without aggression; level 1: one cricket attacks, the other retreats; level 2: antennal fencing; level 3: mandible spreading by one cricket; level 4: mandible spreading by both crickets; level 5: mandible engagement; level 6: grappling, an all-out fight involving repeatedly engagements, biting and tossing. The fight can be concluded at any of the levels 2–6 by one opponent retreating. Fight duration, from first contact until conclusion, was measured to the nearest second with a stopwatch; the duration of any pauses that occasionally occurred when the animals lost contact were deducted.

All statistical analyses were performed using standard commercial software (Prism 5, GraphPad Software Inc. La Jolla, CA, USA) running on a Power Macintosh computer (Apple Computers, Cupertino, CA, USA). The median and the interquartile range (I.Q.R.) were calculated for non-parametric data sets. The Kruskal-Wallis one-way analysis of variance was used to test for significant differences between the three rounds that comprised each tournament. The Mann-Whitney U-test was used to test for significant differences in the distributions between 2 (unpaired) data sets. The Chi-square test was employed for comparing relative frequencies of selected behaviours in 2 groups.
